# Phylogeographic distribution of rhizobia nodulating common bean (*Phaseolus vulgaris* L.) in Ethiopia

**DOI:** 10.1093/femsec/fiab046

**Published:** 2021-03-16

**Authors:** Ashenafi Hailu Gunnabo, Rene Geurts, Endalkachew Wolde-meskel, Tulu Degefu, Ken E. Giller, Joost van Heerwaarden

**Affiliations:** Plant Production Systems Group, Wageningen University & Research, Wageningen, Gelderland, The Netherlands, Postal code: 6708 PB; Laboratory of Molecular Biology, Department of Plant Sciences, Wageningen University & Research, Wageningen, Gelderland, The Netherlands, Postal code: 6708 PB; World Agroforestry Centre (ICRAF), c/o ILRI Campus, Gurd Shola PO Box 5689, Addis Ababa, 4 Ethiopia; International Crops Research Institute for the Semi-Arid Tropics, c/o ILRI Campus, Gurd Shola PO Box 5689, Addis Ababa, Ethiopia; Plant Production Systems Group, Wageningen University & Research, Wageningen, Gelderland, The Netherlands, Postal code: 6708 PB; Plant Production Systems Group, Wageningen University & Research, Wageningen, Gelderland, The Netherlands, Postal code: 6708 PB

**Keywords:** genetic diversity, genospecies, locus, nucleotides, principal coordinate analysis, rhizobial collections

## Abstract

Rhizobia are soilborne bacteria that form symbiotic relations with legumes and fix atmospheric nitrogen. The nitrogen fixation potential depends on several factors such as the type of host and symbionts and on environmental factors that affect the distribution of rhizobia. We isolated bacteria nodulating common bean in Southern Ethiopia to evaluate their genetic diversity and phylogeography at nucleotide, locus (gene/haplotype) and species levels of genetic hierarchy. Phylogenetically, eight rhizobial genospecies (including previous collections) were determined that had less genetic diversity than found among reference strains. The limited genetic diversity of the Ethiopian collections was due to absence of many of the *Rhizobium* lineages known to nodulate beans. *Rhizobium etli* and *Rhizobiumphaseoli* were predominant strains of bean-nodulating rhizobia in Ethiopia. We found no evidence for a phylogeographic pattern in strain distribution. However, joint analysis of the current and previous collections revealed differences between the two collections at nucleotide level of genetic hierarchy. The differences were due to genospecies *Rhizobium aethiopicum* that was only isolated in the earlier collection.

## Teaser

There is a substantial genetic diversity of bean rhizobia but with no local geographic structure in Ethiopia.

## INTRODUCTION

Common bean (*Phaseolus vulgaris* L.) nodulates with soil bacteria, collectively known as rhizobia, that fix atmospheric nitrogen in symbiosis with their host. It is grown across a wide range of environments—at elevations ranging from 800 to 2200 m.a.s.l in Southern Ethiopia (Asfaw 2011) and on a wide range of soil types (Asfaw *et al*. 2009). Compared with other legumes, common bean is believed to be a poor nitrogen fixer under field conditions (Graham 1981) and has been reported to respond erratically to inoculation with elite rhizobia (Martínez-Romero 2003). Poor nitrogen fixing ability and erratic response to inoculation might be related to the promiscuous nature of common bean: it has been shown to establish effective symbioses with taxonomically diverse groups of rhizobia (Martínez-Romero 2003). It is thereby possible that some common bean growing environments or locations lack effective rhizobia, leading to unproductive symbiosis and poor fixation, while localized differences in either competitiveness or effectiveness could lead to varying inoculation responses.

A prerequisite for the above explanation is that geographic or environmental structure exists in populations of common bean-nodulating rhizobia. Geographical structure has been observed in bacteria at the continental (Tian *et al*. 2010; Rashid *et al*. 2014) and regional levels (Wang *et al*. 2012; Cao *et al*. 2014, 2017). Yet some studies show little or no biogeographic patterns among bacteria (Fierer and Jackson 2006), supporting the theory that in terms of bacterial diversity ‘everything is everywhere—the environment selects’ (Martiny *et al*. 2006). The latter theory would still predict geographic structure in bacterial populations, but only where environments differ.

Several environmental variables such as pH, moisture, salinity, nitrogen, temperature, elevation and latitude have been reported to influence the biogeographic structure in rhizobium (Van Cauwenberghe, Michiels and Honnay 2015; Stefan *et al*. 2018; Zhang *et al*. 2018). For instance, an apparent shift in the relative dominance of rhizobial types with soil pH was shown in tropical tree legumes such as *Gliricidia sepium*, *Calliandra calothyrsus*, *Leucaena leucocephala* and *Sesbania sesban* (Bala and Giller 2006). With common bean, *R. tropici* and *R. leucaenae* strains were found to dominate in acid soils (Anyango *et al*. 1995; Baginsky *et al*. 2015), *R. etli* strains in slightly acidic to moderately alkaline soils, while *R. leguminosarum* strains seem to be most common on neutral soils (Baginsky *et al*. 2015). Additionally, the composition of sampled rhizobia may vary spatially, temporally and as a function of plant genotype (Zhang *et al*. 2011; Fan *et al*. 2017; Burghardt 2020). Such genetic differentiation between populations of rhizobia may consequently contribute to variations in terms of symbiotic effectiveness and response of common bean to inoculation.

Studies of biogeographic structure in bacteria have been done at community (Bissett *et al*. 2010; Zhang *et al*. 2018), species (Zhang *et al*. 2011; Adhikari, Itoh and Suyama 2013; Baginsky *et al*. 2015) and molecular levels, with the latter ranging from electrotypes (Pinero, Martinez and Selander 1988), different loci on a chromosome (Silva *et al*. 2005), haplotypes (Cao *et al*. 2017) to sequence type levels (Stefan *et al*. 2018). In terms of relevance to symbiotic effectiveness, it is hard to define the most appropriate level for describing genetic diversity. Although certain rhizobial species may be associated with effective nodulation in specific legumes, symbiotic effectiveness is known to vary within bacterial species (Thrall, Burdon and Woods 2000; Thrall *et al*. 2011; Dwivedi *et al*. 2015). At the molecular level, it is known that symbiotic genes, which in most fast-growing rhizobia are located on a plasmid, may be transmitted horizontally between strains and species. This suggests that the genetic determinants of compatibility and effectiveness may be structured differently from other genes and their patterns of diversity may not be well described at species level. It thereby follows that studies of geographic structure should be done at different levels of taxonomic hierarchy, such as at nucleotide, locus (haplotype) or species levels, something that is not always done in practice. In addition, molecular and statistical tools with poor resolution of species delineation (Laguerre *et al*. 2003; Tian *et al*. 2010) may affect the predictions of rhizobial geographic structure (Stefan *et al*. 2018). To minimize this bias, multilocus sequence analysis (MLSA) together with symbiotic gene (*nifH* and *nodC*) analysis was suggested for better description of taxonomic and genetic diversity.

Here, we present a study that aims to establish the occurrence of biogeographic structure in bean-nodulating rhizobia in Ethiopia using multilocus and symbiotic gene sequence analysis. By combining 72 newly sampled strains with published data on 27 strains sampled over a wider area (Aserse *et al*. 2012), we are able to describe patterns of diversity at different levels of geographic and taxonomic hierarchy. We thereby evaluate the levels and patterns of genetic diversity present in Ethiopia and test whether taxonomic composition and sequence diversity are associated with geographic distance, environmental factors such as pH and elevation and whether they are consistent between independent samples taken at different times and spatial scales.

## MATERIALS AND METHODS

**Figure 1.**
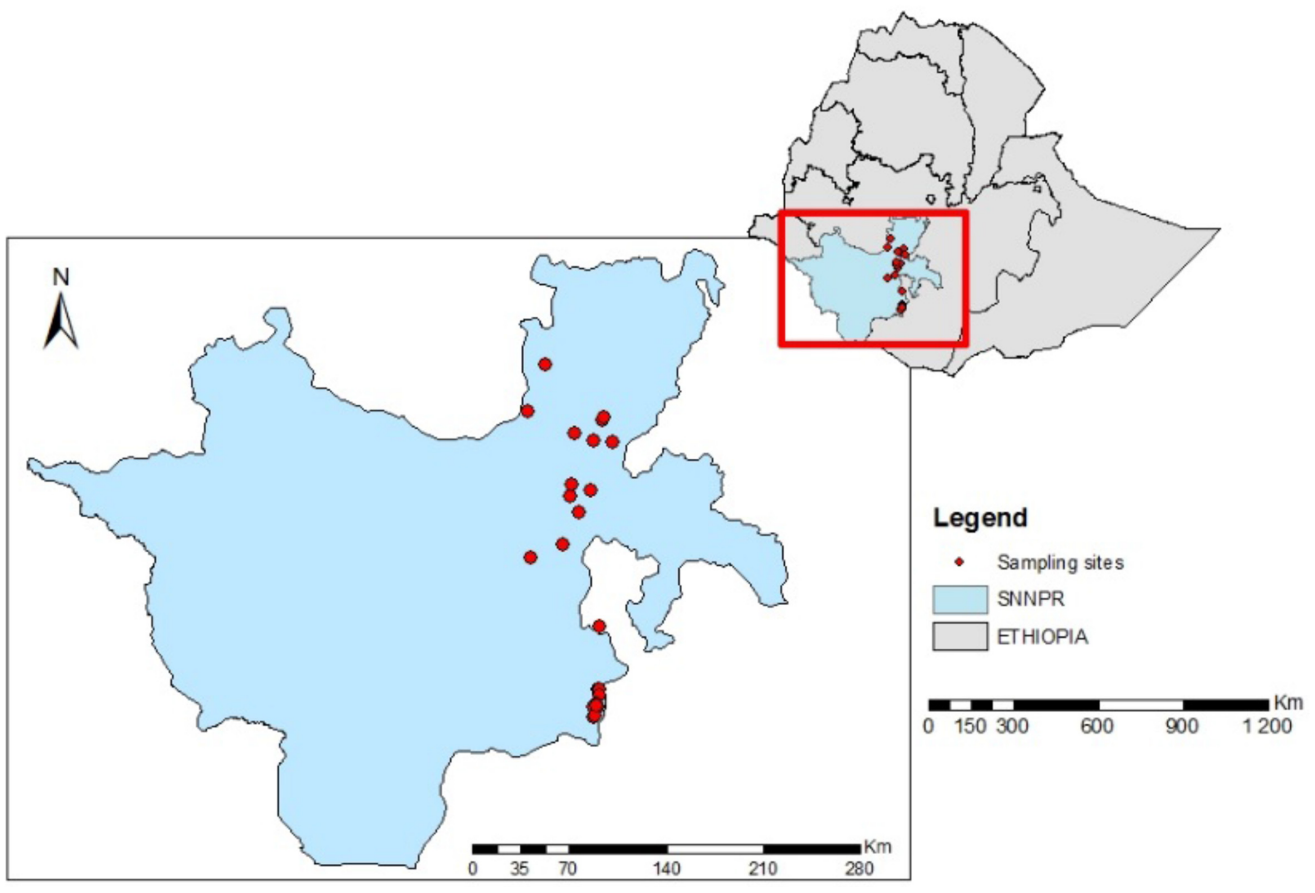
Soil sampling sites in Southern Ethiopia.

In March 2016, soil samples were collected from 18 locations where common bean is grown in Southern Ethiopia with no history of previous rhizobium inoculation (Fig. 1; Table S1, Supporting Information), following procedures described previously (Wolde-meskel *et al*. 2004). Some nodules were also collected from standing bean plants in May 2016 and maintained in glass vials containing silica gel. The collected soil samples and nodules were immediately transported to Hawassa College of Agriculture for trapping and rhizobia isolation. Three-kilogram-capacity pots, pre-treated with 96% ethanol, were filled with each of the soil samples. Three locally released and well-adapted common bean cultivars, namely *Hawassa dume*,*Nasir* (Mesoamerican genotypes) and *Ibado* (Andean genotype), were used to trap rhizobia from the soil samples by considering that the varieties would reveal preferential selection of strains due to host genotype × strain interactions. The seeds of the selected varieties were surface sterilized in 96% ethanol for 1–2 min and then rinsed in 4% sodium hypochlorite for 4 min. They were then washed in several changes of sterile water. The surface-sterilized seeds were germinated in sterile Petri dishes on sterile tissue paper.

The germinated seedlings were aseptically transplanted into the pots containing the soil samples. Each of the experimental units was arranged in a completely randomized block design with three replications. The seedlings were carefully monitored, watered with sterile water as needed and allowed to grow until the early flowering stage. At the early flowering stage, the plants were carefully uprooted, and the roots were washed in tap water. Roots containing bigger and healthier nodules were sorted, and the nodules were carefully excised and maintained in plastic vials containing dry silica gel and cotton. The vials were preserved in a refrigerator at 4°C until used to isolate rhizobia (Somasegaran and Hoben 1994) and the set of strains isolated from these nodules is referred to here as the ‘current rhizobia collection’. An additional set of published rhizobial sequence data was used, representing strains isolated in 2007 from local Ethiopian bean cultivars and from root nodules of the *Redwolayta* bean cultivar grown in pots on soils from Addis Ababa (Aserse *et al*. 2012), and is referred to here as the ‘previous rhizobia collection’.

### Isolating rhizobia from nodules

A total of 89 rhizobia strains were isolated from common bean root nodules grown in pots on soil samples collected from various locations and/or nodules collected from field standing beans in Southern Ethiopia (Table S1, Supporting Information). The desiccated nodules were rehydrated by imbibing in sterile water for overnight. The imbibed nodules were surface sterilized by rinsing in 96% ethanol for 20–30 s and then in 4% sodium hypochlorite for 4 min and rinsed in six changes of sterile distilled water (Somasegaran and Hoben 1994; Howieson and Dilworth 2016). The surface-sterilized nodules were crushed in a sterile test tube containing a drop of normal saline solution. A loopful of nodule suspension was then serially streaked on yeast-extract mannitol agar (YMA) containing 25 µg/ml Congo red dye. The streaked Petri plates were incubated for 5–13 days, and daily assessed for colony appearance. A single colony was picked and further streaked on new YMA several times to obtain pure colonies. We further streaked the isolates on YMA containing 25 µg/ml bromothymol blue and on pentose glucose agar containing 25 µg/ml bromocresol purple.

### Soil analysis

At the time of nodule and soil sample collection for trapping rhizobia, 200 g of soil sample from each site was collected in separate plastic bags, dried and maintained in a clean and dry area before determining soil physicochemical parameters at Horticoop, Addis Ababa, Ethiopia. Soil acidity (pH) was determined by pH-H_2_O method, organic carbon by Walkley and Black (1934), soil total nitrogen by Kjeldahl (Bremner 1960) and available phosphorus by Olsen's method (Olsen *et al*. 1954). Futhermore, cation exchange capacity was extracted in ammonium acetate (Bache 1976), calcium, potassium, magnesium and sulfur in Mehlich-3 extraction (Mehlich 1984); and copper, iron, manganese, zinc and boron in diethylenetriamine penta-acetic acid (DTPA) extraction (de Abreu *et al*. 1998).

### Molecular characterization and phylogenetic analysis

We characterized the current rhizobia strains genetically using the primers listed in Table S2 (Supporting Information). All genotyping procedures, including DNA sequencing, sequence trimming and alignment were reported previously (Gunnabo *et al*. 2019). For multilocus analyses, sequence alignments of the full set of strains (25 previously characterized and 72 currently isolated strains) were concatenated in R 3.6.1 (R Core Team 2020). Best fitting substitution models for concatenated sequences (16S, *glnII*, *recA*, *rpoB*) and symbiotic genes (*nodC* and *nifH*) were identified using maximum likelihood (ML) procedures in MEGA 7. Substitution models with lower BIC (Bayesian information criterion) and AICc (Akaike information criterion corrected) values were selected for construction of phylogenetic trees. As a result, the maximum likelihood phylogeny of the concatenated sequences was constructed in R using the general time reversible (GTR) model with gamma distributed rates (+G) with presence of invariant sites (+I); phylogeny of 16S rRNA using Kimura-2-parameter model with +G, phylogeny of *nifH* using Tamura 3-Parameter model with +G; and phylogeny of *nodC* using Tamura 3-Parameter (T92) model with +G+I. The robustness of the tree topologies was evaluated using bootstrap (BT) analysis with 100 replications under the ML statistical method using *ape* package in R. Furthermore, pairwise average nucleotide identity (ANI%) was calculated for sequence types within and between clusters as we did previously (Gunnabo *et al*. 2019).

Phylogenetic congruence of individual loci with respect to a full, multilocus alignment was evaluated by comparing the log-likelihood of fully optimized gene-specific phylogenies optimized with that for phylogenies with the topology constrained to the maximum-likelihood topology inferred for the full alignment (Planet 2006). Significance of the log-likelihood difference was evaluated by parametric bootstrap.

### Assigning reference species to monophyletic clades

After reconstructing the phylogenetic trees, clade support was calculated based on tree bipartition (Efron, Halloran and Holmes 1996) using the *boot.phylo()* function from *ape* package in R. Clade identity for Ethiopian strains was assigned by identifying the smallest, well-supported (bootstrap value >60%) monophyletic clade containing at least one references species. In case no such clade was identified, strains were assigned to their own clade. The named and unnamed clades (genospecies) thus identified were used as a basis for calculating clade-level diversity measures (see below).

### Diversity and phylogeographic analysis

Genetic diversity was calculated at different levels of genomic hierarchy (Table S3, Supporting Information). At the species level, Shannon and Simpson indices (Hill *et al*. 2003) were calculated based on genospecies using the vegan package in R. At the level of individual genes, allele sharing distance was calculated and averaged across loci, while at the nucleotide level, the pairwise proportion of different sites was calculated (Nelson and Hughes 2015).

Patterns in biogeographic distribution of the rhizobia were estimated at nucleotide, locus and species levels using the Mantel test, which evaluates the relationship between geographic distance and genetic divergence (Diniz-Filho *et al*. 2013) using packages in R. Partial Mantel tests (Diniz-Filho *et al*. 2013) were used to test for association of rhizobial distribution with geographical covariates. Pairwise relatedness among individual rhizobial strains as a function of geographic distance were visualized by principal coordinates analysis (PCoA) (Gower 1966). Genetic distances between the strains and the geographic distances were decomposed into independent genetic and geographic dimensions, respectively, and the first component for each was plotted against each other.

To describe the dependence of genetic diversity as a function of distance, a sliding-window resampling analysis was performed. Windows of 80 km width were moved at 10 km intervals, assuming only few loci (haplotype) dominate within this distance (Hollowell *et al*. 2016). For each interval, diversity statistics were calculated for a randomly selected pair of strains within the specified distance interval. Genetic diversity values of strains sampled at random distances were calculated for comparison.

Nucleotide sequences of the strains studied here have been deposited in the GenBank (https://www.ncbi.nlm.nih.gov/) with accession numbers MT380934–MT381020 (16s rRNA), MT381021–MT381097 (nodC), MT381098–MT381180 (glnII), MT381181–MT381268 (gyrB), MT381269–MT381357 (recA), MT381358–MT381440 (rpoB) and MT381441–MT381517 (nifH).

## RESULTS

### Phylogenetic analysis

A total of 72 newly isolated and 23 previously collected strains were used for phylogenetic analysis. Alignments of 149 (72 current, 23 previous and 54 reference) sequence types obtained from 16S rRNA (548 base pairs, bp), *glnII* (461 bp), *recA* (349 bp) and *rpoB* (784 bp) were concatenated and yielded 2142 bp positions (Table 1) and analyzed phylogenetically. The lengths of sequence alignments used in the concatenation were trimmed to match with previous and reference sequences that resulted in a significantly smaller bp size than those obtained from sequencing. Incongruence tests between the individual genes and the full, multilocus alignment showed all loci to be congruent with the multilocus phylogeny, with the exception of *nodC*, for which the log likelihood for the optimized topology was substantially higher than for the reference topology. It was therefore decided to perform subsequent phylogenetic and diversity analysis separately for the four concatenated housekeeping genes and *nodC*.

**Figure 3.**
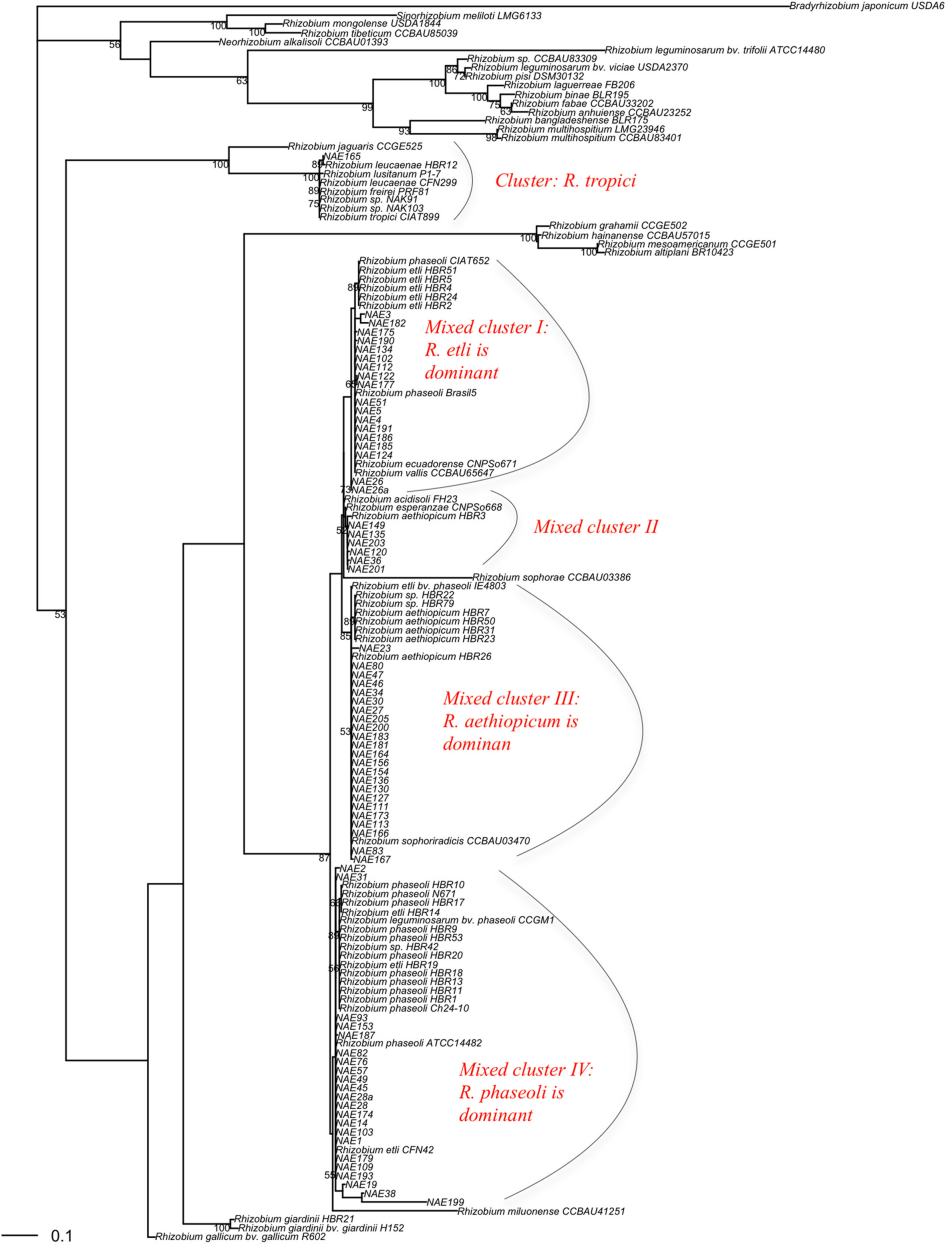
Evolutionary phylogeny of symbiotic (nodC) gene reconstructed based on T92 + G + I nucleotide substitution models. Description of the codes of the strains in the phylogeny is shown in previous Fig. 2.

**Figure 5.**
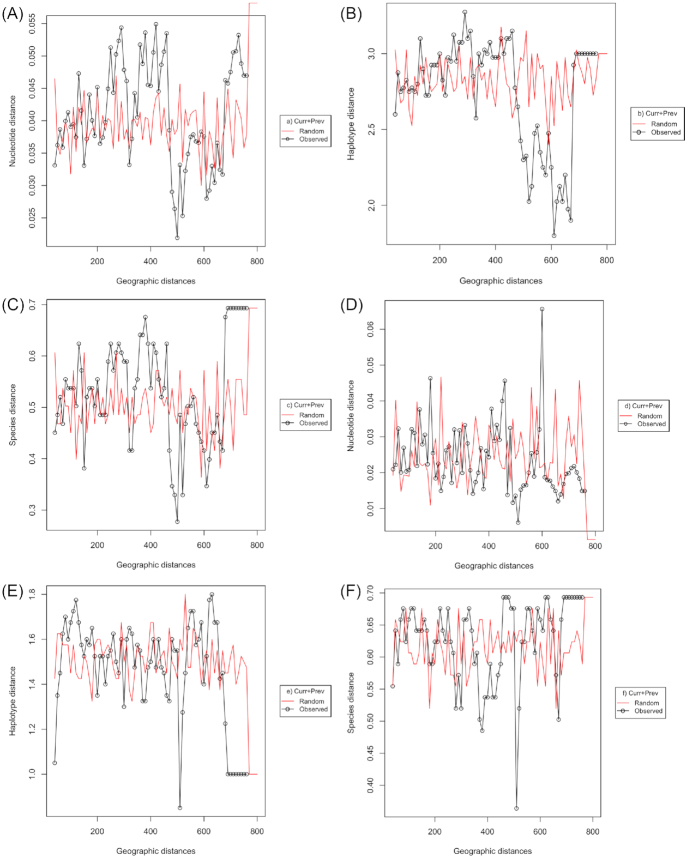
Dependency of rhizobia distribution on geographic distances. The circular dots connected by red lines show a randomly sampled hypothetical rhizobia in a sliding window of 80 km, whose genetic distance was estimated at every 10 km interval. The circular dots connected by black lines show genetic distances of the actual rhizobia (observed) samples. Divergency between the random (hypothetical) and the observed samples over the distances indicates non-random variation and reflects dependency of genetic distribution on geography, while overlapping indicates no genetic dependency on the geography. The genetic distances were estimated at **(A)** nucleotide **(B)** haplotype and **(C)** species levels using HK genes of both current (Curr) and previous (Prev) rhizobia collections. Similarly, genetic distances of symbiotic (Sym) genes are plotted at **(D)** nucleotide **(E)** locus/haplotype and **(F)** species levels. The genetic and geographic distances are plotted on the *y*-axis and *x*-axis, respectively.

**Table 1.** Gene locus alignment statistics calculated in MEGA 7.

Gene locus	No. of sequences	No. of alignment sites	No. of conserved regions	No. of variable sites	No. and percentage of parsimony informative sites	No. of singletons
^a^16S rRNA	188/42U	548	450	91	41	50
*glnII*	184/96U	461	260	201	169	32
*recA*	190/96U	349	191	154	144	10
*rpoB*	166/100U	784	362	362	326	36
Concatenated	147/117U	2142	1346	783	597	186
*nodC*	157/46U	465	162	301	263	38
*nifH*	158/61U	312	136	167	142	25

U = Unique taxes maintained by DAMBE program for each gene locus from the total sequences used for this analysis. Concatenated stands for combined genes 16S rRNA + *glnII* + *recA* + *rpoB*. The alignment statistics was calculated from all sequences obtained from current, previous and reference strains.

a Numbers of the sequence alignment sites for 16S rRNA are reduced due to trimming to match with reference strains.

### Multilocus sequence analysis

**Figure 2.**
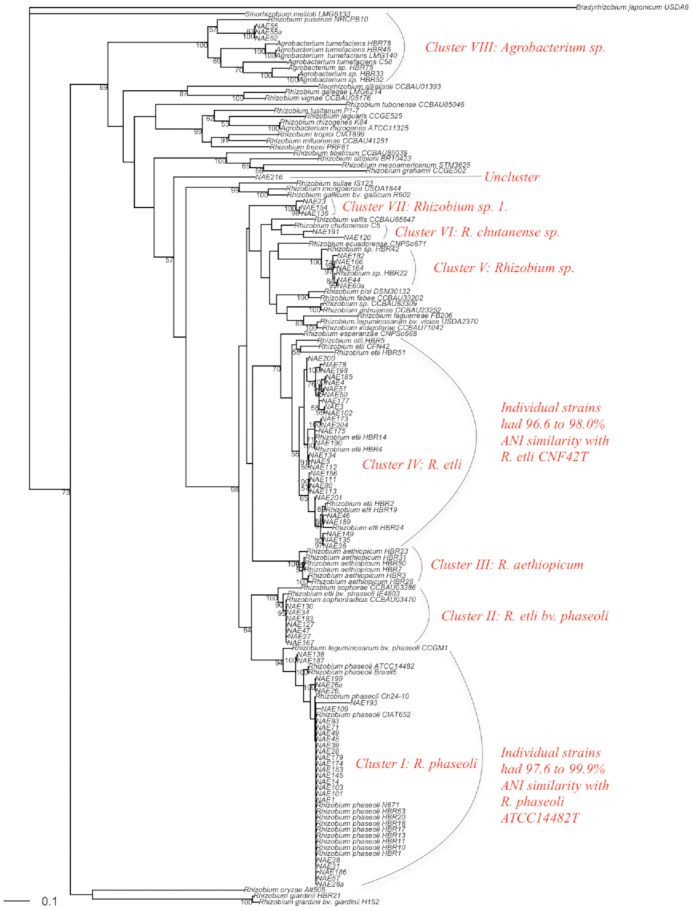
A maximum likelihood evolutionary phylogeny of bean rhizobia reconstructed based on MLSA of concatenated 16S rRNA, *glnII*, *recA* and *rpoB* genes. The evolutionary history was inferred using the GTR model with gamma distribution (+G) and invariant rates among sites (+I) under maximum likelihood statistical method using ape package in R 3.6.1. The robustness of tree topology was optimized by 100 replications of BT analysis. Strains with HBR names indicate the previous collection; NAE indicate the current collections and the others are reference strains.

The phylogeny of the concatenated housekeeping gene sequences (MLSA) grouped the current and previous collections of strains into seven *Rhizobium* and one *Agrobacterium* lineages (Fig. 2) designated as cluster I–VIII, hereafter regarded as genospecies for clarity, and one ‘Unclustered’ strain NAE216. The first cluster (Cluster I) of the *Rhizobium* lineage (genospecies) was the second largest cluster that comprised 36% of the current and 34.7% of the previous isolates. Strains in this cluster shared 97.6–99.9% ANI with the type strain of *R. phaseoli* ATCC 14482. In this analysis, a reference strain CIAT 652 that was recently reclassified as *R. phaseoli* (Díaz-Alcántara *et al*. 2014) was also assigned to this cluster. The cluster also contained other *R. phaseoli* strains Brasil5, Ch24-10 and N671 and *R. leguminosarum* bv. *phaseoli* (*Rlp*) CCGM1. The *Rlp* strain CCGM1 was found to form a monophyletic group with cluster I, showing their close relatedness to *R. phaseoli* strains.

Strains within genospecies II were phylogenetically related to *R. etli* bv. *phaseoli* IE4803, and to the type strains of *R. sophorae* CCBAU 03386 and *R. sophoriradicis* CCBAU 03470 with 100% BT support, signaling some weakness of MLSA to clearly delineate these species. None of the strains of Ethiopian origin reported in previous study (Aserse *et al*. 2017) was related to this genospecies, indicating differences between the two strain collections. The third genospecies (genospecies III) consisted of strains only from previous collection and assigned with *R. aethiopicum* HBR26^T^, which was isolated from root nodule of common bean growing in Ethiopia (Aserse *et al*. 2012, 2017). The absence of strains belonging to the species *R. aethiopicum* from the current collection could reflect either the species is confined to certain biogeographic location or sensitive to sampling strategies.

The fourth cluster (Cluster IV) of the *Rhizobium* lineage is the largest cluster that comprised 37.5% of current and 30% of the previous strain collections. The cluster was assigned with type strain *R. etli* CFN42 with lower BT value. The strains in this cluster shared 96.6–98.0% ANI or sequence similarity with *R. etli* CFN42^T^ and considered *R. etli* strains according to the threshold level of criterion specified for species delineation (>95% ANI) previously (Goris *et al*. 2007).

The clusters V, VI and VII of the *Rhizobium* genospecies belonged to *Rhizobium* sp. complex that were recently considered as *R. leguminosarum–etli* clade (Young *et al*. 2021). The fifth genospecies (genospecies V) of this complex category consisted strains that were previously reported as *Rhizobium* sp. (Aserse *et al*. 2012). They were also closely related to *R. ecuadorense* CNPSo671^T^ with a lower bootstrap support, while strains in cluster VI (which contained only two strains NAE120 and NAE191) belonged to *R. chutanense* C5^T^ strain that was recently isolated from root nodule of common bean grown in China (Huo *et al*. 2019). The strains NAE120 and NAE191 share 95.5% and 97.2% sequence similarity with *R. chutanense*, respectively. *Rhizobium chutanense* is in turn closely related to *R. vallis* CCBAU 65647^T^ with which NAE120 shares 93.8% while NAE191 shares 95.0% ANI. Hence, we concluded that the strain NAE120 and NAE191 can be considered to belong to the *R. chutanense* genospecies. The VII cluster contained three strains (NAE23, NAE36, NAE154) that did not belong to any refence or type strain. They could represent a new genospecies that is tentatively named as *Rhizobium* sp. 1. There was also a single unclustered strain NAE216 that we isolated from a common bean root nodule. This strain may remain unidentified until a closely related strain is isolated and characterized.

In the current analysis, we retrieved *Agrobacterium* strains from bean root nodules, but a reinfection test showed these were unable to induce nodules on the host plant. These strains were included within genospecies VIII and shared 100% BT support with *Agrobacterium tumefaciens* C58^T^. About 18% (from previous collection) and 4% (the test strains in this study) belong to this group, suggesting that *Agrobacterium* sp. frequently enter the root nodules of common bean.

Overall, the majority (∼70%) of the strains belong to *R. etli* and *R. phaseoli*, confirming the previous report that they are dominant strains in Ethiopian soils (Aserse *et al*. 2012). *Rhizobium etli* was relatively more diverse (with nucleotide diversity of 0.014) than *R. phaseoli* (with nucleotide diversity of 0.005; Table 2). Bean is infrequently nodulated by *R. tropici* strains in Ethiopian soils since only a single strain NAE165 was assigned to be *R. tropici* based on symbiotic (Fig. 3) and other protein coding housekeeping genes. Among the strains of Ethiopian origin from previous collection (Aserse *et al*. 2017), *R. leucaenae* HBR12 was the only strain related to *R. tropici*, confirming its rarity. The strains NAE165 and HBR12 were not included in the current MLSA phylogeny due to lack of sequences for the *rpoB* gene. Similarly, it can be said that *R. giardinii* (now renamed as *Pararhizobium giardinii*; Mousavi *et al*. 2014) strains occur only occasionally in root nodules of beans growing in Ethiopia as far as no such strain was described from the current collection, but only a single strain was reported previously (Aserse *et al*. 2012).

**Table 2.** Nucleotide and locus diversities of strains per species or clusters on the basis of concatenated housekeeping (HK) and symbiotic gene nodC analysis.

	HK gene diversity (genospecies)			Sym gene diversity (symbiovars)		
Genospecies/symbiovars	No.	Nucl	Locus	No.	Nucl	Locus
*R. phaseoli* ATCC 14482	31	0.005	0.671	27	0.0000	0.000
*R. phaseoli Brasil5*	NA	NA	NA	22	0.0000	0.000
*R. etli* CFN42	34	0.014	0.986	NA	NA	NA
*R. ecuadorense/Rhizobium*sp.	8	0.015	0.964	NA	NA	NA
*R. sophorae* CCBAU03386	NA	NA	NA	7	0.00133	0.417
*Rep/R. sophoriradicis*	7	0.001	0.786	29	0.00052	0.061
*R. aethiopicum*	6	0.005	0.933	NA	NA	NA
*Rhizobium leucaenae CFN299*	NA	NA	NA	4	0.0000	0.000
*Unclustered group*	2	0.024	1.000	NA	NA	NA
*Rhizobium*sp. I.	3	0.004	1.000	NA	NA	NA

HK stands for housekeeping, Sym stands for symbiotic, No. stands for number, Nucl stands for nucleotides and *Rep* stands for *Rhizobium etli* bv. *phaseoli*.

### Symbiotic genes *nifH* and *nodC*

Individual trees reconstructed from *nifH* and *nodC* gene sequences resulted in a mixture of species that appeared within distinctly different clusters of MLSA phylogeny. The *nifH* and *nodC* genes are incongruent genes showing conflicting evolutionary histories. Hereafter, the strains in clusters formed after the symbiotic gene analysis are named *symbiovars* (Rogel *et al*. 2011). Since the *nifH* gene is highly conserved, its phylogeny clustered most of the strains into a single clade and is excluded henceforth. A total of 157 (77 current, 27 previous and 53 reference) sequences (Table 1) of *nodC* (465 bp) were subjected to the phylogenetic analysis. The symbiotic gene *nodC* is also incongruent with the housekeeping genes and resulted in quite different tree topology supported with lower internal BT values. The *nodC* phylogeny formed five symbiovar clusters, in which the first four clusters supported with 100% outer BT value and each cluster consisted strains belonging to different species. Consequently, the first four symbiovars were recognized as mixed clusters I–IV (Fig. 3). The *R. aethiopicum* genospecies now appeared as dominant symbiovars of mixed cluster III including other reference species such as *R. sophoriradicis* and *R. etli* bv. *phaseoli*. The fifth cluster belongs to reference species *R. tropici* (Cluster: *R. tropici*) that included the Kenyan strains NAK91 and NAK103 and the newly isolated strain NAE165 from Ethiopia. Cross-tabulation of the symbiovars and genospecies revealed that genospecies I (Cluster I) largely corresponds to the symbiovar mixed cluster IV, both of which are closely related to type strain *R. phaseoli* ATCC 14482^T^. The *R. etli* strains of the genospecies cluster IV correspond to symbiovar I in the *nodC* phylogeny that belong to reference strain *R. phaseoli* Brasil5 (Table 3). The *Rlp* strain CCGM1 that was found to group closely with the *R. phaseoli* genospecies now shared similar symbiotic gene nodC with *R. phaseoli* strain. The complexity of the mixed clusters may reflect the close relatedness of the strains as also shown by the MLSA among which we expected higher genetic exchange. In general, the *nodC* phylogeny clearly separated *R. tropici* and the *Rhizobium* complex *(R. phaseoli–R. etli–Rhizobium* sp.) lineages. The *Agrobacterium* strains did not possess the *nodC* gene and were not part of the phylogeny. Since the *nodC* (symbiotic) phylogeny had poor resolution, we used both the symbiotic (Sym) and housekeeping (HK) gene phylogenies to evaluate the phylogeography of common bean rhizobia.

**Table 3.** Cross-tabulation of genospecies and symbiovars.

	Symbiovars (nodC clusters)									
		*R. giardinii* H152 (U)	*R. lentis* BLR27 (V)	*R. leucaenae* CFN299 (VI)	*R. phaseoli* ATCC 14482 (IV)	*R. phaseoli* Brasil5 (I)	*R. sophorae* CCBAU03386 (II)	*R. sophoriradicis* CCBAU03470 (II)	Unclustered symbiovars (UK)	Total
Genospecies (HK)	*Rhizobium*sp. (V)	0	0	0	1	1	0	3	0	5
	*R. etli* CFN42 (IV)	0	1	0	2	18	5	8	0	33
	*R. giardinii* bv. *giardinii* H152 (U)	2	0	0	0	0	0	0	0	2
	*R. phaseoli* ATCC 14482 (I)	0	3	0	26	3	0	0	2	34
	*R. sophoriradicis* CCBAU03470 (II)	0	0	0	0	0	0	8	0	8
	Unclustered genospecies	0	0	0	0	1	1	0	0	2
	Rhizobium sp. I (VI)	0	0	0	0	0	0	3	0	3
	*R. aethiopicum HBR26* (III)	0	0	0	0	0	1	5	0	6
	*R. leucaenae* CFN299 (no in HK)	0	0	4	0	0	0	0	0	4
	Total	2	4	4	29	23	7	27	2	98

HK stands for housekeeping genes, U stands for unclustered and Roman numbers in parenthesis indicate cluster (genospecies) numbers.

We thus inspected population abundance and genetic diversity within genospecies or symbiovars (Table 2) and found that the two dominant species (*R. etli* and *R. phaseoli*) were equally abundant in Ethiopian soils. Regarding genetic diversity, *R. etli* was more diverse than *R. phaseoli* at nucleotide and locus levels for the genospecies. However, for the *nodC* gene, different symbiovar clusters were obtained in which case *R. etli* strains now became part of the *R. sophorae* and *R. sophoriradicis* symbiovar clusters (Table 3).

### Genetic diversity analysis

In the current analysis, the reference strains included were selected to represent the widest possible range of rhizobia, and can be considered as providing a benchmark for maximum levels of genetic diversity (Table 4). Not surprisingly therefore, both the current and previous collections of rhizobia had significantly less genetic diversity in terms of housekeeping and symbiotic genes when compared with the entire set of reference strains (*P* < 0.001). The reduction in genetic diversity was less severe when judged against the set of reference strains known to nodulate common bean but was still considerable, and was mostly due to the absence from Ethiopia of some strains known to nodulate common bean, such as *R. galegae*,*R. gallicum*,*R. anhuiense* and *R. mesoamericanum*. There was a further reduction in *nodC* gene diversity when comparing the reference strains to the Ethiopian sample as a whole, reflecting the very low degree of within-clade diversity observed for this gene (Table 3).

**Table 4.** Comparison of genetic diversity of bean-nodulating rhizobial strains.

		Housekeeping genes			
	Type of strains	Nucleotide	Locus	Shannon	Simpson
Genospecies					
	^a^All references	0.085	0.999	3.433	0.958
	^b^All Ref. in Eth. bean clusters	0.046	0.994	2.379	0.864
	^c^Bean-nodulating references	0.071	0.997	2.791	0.914
	^d^Bean-nodulating references in the current clusters	0.043	0.987	1.631	0.734
	^e^Previous strains	0.061	0.924	1.628	0.787
	^e^Current strains	0.042	0.965	1.556	0.726
	^f^Ethiopian	0.048	0.956	1.731	0.762
Symbiovars					
	^a^All references	0.232	0.979	3.408	0.960
	^b^ All Ref. in Eth. bean clusters	0.128	0.926	2.248	0.880
	^c^Bean-nodulating references	0.207	0.959	2.808	0.926
	^d^Bean-nodulating references in the current clusters	0.128	0.926	2.248	0.880
	^e^Previous strains	0.048	0.801	1.677	0.781
	^e^Current strains	0.039	0.796	1.672	0.762
	^f^Ethiopian	0.041	0.794	1.799	0.781

a All of the reference strains that were used in symbiotic and housekeeping gene analysis.

b All of the reference strains (both bean-nodulating and non-bean-nodulating references) that were only grouped within monophyletic clusters containing local strains.

c All of the bean-nodulating references that are either within or outside of the monophyletic clusters containing the local strains.

d Some of the only bean-nodulating reference strains that were grouped within the clusters containing the local strains.

e Local strains collected from Ethiopia (previous and current strain collections).

f Ethiopian strains include the previous and current collections.

Strains from the previous collections were more diverse than the current collections at nucleotide, locus and species levels both for symbiotic and housekeeping genes (Table 2). The latter is somewhat surprising given that the previous collection (Aserse *et al*. 2012) represented a much wider geographic area than the current sample and would suggest an absence of geographic structure, something we address in more detail below.

### Genetic structure and phylogeography of rhizobia

**Figure 4.**
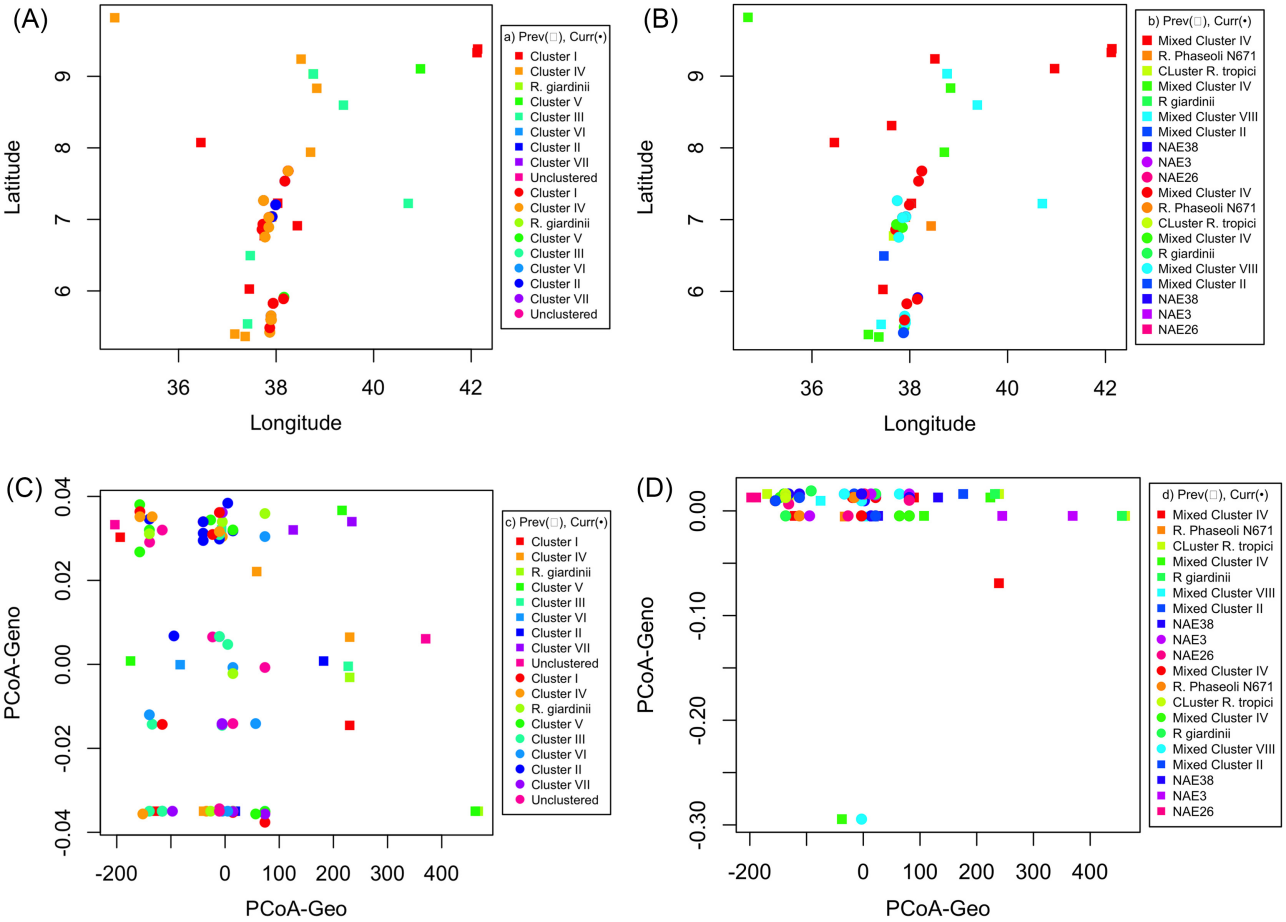
Distribution of genospecies and symbiovars in plane and PCoA. **(A)** Distribution of genospecies. **(B)** Distribution of symbiovars. **(C)** PCoA of genospecies and geographic distances. **(D)** PCoA of symbiovars and geographic distances. ‘Prev.’ = previous strains; ‘Curr.’ = current strains. The clusters in the legend represent the genetic groups of the rhizobial isolates based housekeeping gene phylogeny (a, b) and symbiotic gene nodC phylogeny (b, d). The PCoA-Geo represents geographic coordinates, while the PCoA-Geno represents the genetic distances between the strains. Rectangular dots represent strains from previous (Prev) culture collection and circular dots indicate strains from current (Curr) culture collection.

The two collections differed significantly in terms of genospecies composition (Fisher’s exact test *P* < 0.00292), a fact that turned out to be entirely due to the absence of the previously identified genospecies *R. aethiopicum* in the current sample. Removal of the *R. aethiopicum* clade led to a loss of significance of Fisher’s exact test (*P* = 0.364). The absence of *R. aethiopicum* in the current sample is noteworthy, since the genospecies was found throughout the sampling area, including the same region (Fig. 4A) as where the current sample was taken. The difference in genospecies composition between the two collections was influenced by soil pH since the previous collection was obtained from soils with considerably higher pH values (6.0–8.78) compared with the soils sampled for the current collection (pH 4.68–6.44).

It is interesting to note that at the level of *nodC* symbiovars, a large proportion of the current sample was actually assigned to the same clade (associated with *R. sophoriradicis*) as *R. aethiopicum*, which was almost equally common as the *R. etli* and *R. phaseoli* symbiovars. In fact, there was no significant difference in symbiovar composition between the two samples. This suggests that a uniquely Ethiopian symbiovar exists that is associated, albeit not uniquely, with *R. aethiopicum*.

Mantel tests and partial mantel tests did not reveal any significant association of genetic distance with either geographic distance, elevation or pH, particularly after correcting for collection (Table 5). pH influences distribution of the strains when sample collections were analyzed jointly (Table 6), but disappeared when analyzed separately. Similarly, distribution of genospecies or symbiovars relative to all others, PCoA analysis (Fig. 4) and sliding window analysis of genetic diversity as a function of geographic distance (Fig. 5) did not reveal any obvious pattern, except some random distribution of samples at nucleotide and species levels for housekeeping genes and at nucleotides for symbiotic genes (Fig. 5A, C and D). In short, we argue that there is no spatial pattern in the rhizobial population.

**Table 5.** Genetic distribution of rhizobia across space at nucleotide, haplotype and species.

Genetic distance matrices (D)			Sign. statistics (HK)		Sign. statistics (Sym)	
Matrix D1	Matrix D2	Cov.D3	r_(partial/Mantel)_	*P*	r_(partial/Mantel)_	*p*
Nucleotides	Geo		0.085	0.073.	−0.021	0.468
	Source		0.085	0.036*	0.094	0.114
	pH		−0.034	0.837	0.05	0.127
	Alt		0.076	0.072.	−0.0002	0.342
	Geo	Source	0.053	0.131	−0.066	0.865
	Geo	pH	0.085	0.082.	−0.020	0.431
	Geo	Alt	0.058	0.131	−0.023	0.524
	Alt	Source	0.051	0.133	−0.033	0.591
	Alt	Source	−0.042	0.895	0.051	0.152
Haplotypes	Geo		−0.026	0.621	−0.036	0.856
	Source		−0.019	0.649	0.006	0.377
	pH		−0.010	0.570	−0.006	0.589
	Alt		0.011	0.402	−0.047	0.960
	Geo	Source	−0.019	0.589	−0.042	0.933
	Geo	pH	−0.026	0.676	−0.036	0.884
	Geo	Alt	−0.033	0.728	−0.017	0.695
	Alt	Source	0.018	0.355	−0.051	0.983
	Alt	Source	−0.009	0.566	−0.006	0.577
Species	Geo		0.031	0.230	−0.022	0.750
	Source		0.047	0.097.	0.019	0.217
	pH		−0.017	0.707	−0.001	0.483
	Alt		0.002	0.470	−0.034	0.882
	Geo	Source	0.012	0.346	−0.033	0.908
	Geo	pH	0.031	0.208	−0.022	0.752
	Geo	Alt	0.034	0.211	−0.008	0.568
	Alt	Source	−0.015	0.660	−0.043	0.953
	Alt	Source	−0.021	0.753	0.002	0.471

pH = distance matrix between low pH and high pH communities of rhizobia, Geo = geographic distance, Source = distance matrix between the two sources of strain collections, Alt = distance matrix based on altitude, ‘Cova. D’ = covariance distance matrix for partial Mantel test, Sign. = significance, r_(partial/Mantel)_ = coefficient of association for Mantel or partial Mantel tests, HK = housekeeping genes, Sym = symbiotic genes. Significance levels: ***P < 0.001; **P < 0.01; *P < 0.05; ‘.’,*P* < 0.1.

**Table 6.** Influence of environmental factors on biogeographic distribution of bean-nodulating rhizobia.

Source of strains	Source of variations	Df	χ^2^	*F*	Pr (>F)
Previous + current	Long	1	0.093	1.365	0.24
	Lat	1	0.061	0.897	0.395
	Alt	1	0.072	1.065	0.375
	pH.H_2_O	1	0.219	3.230	0.01*
	Residuals	29	1.964		

χ^2^: Chi-square, Significance codes: ‘***’ 0.001; ‘**’ 0.01; ‘*’ 0.05; ‘.’ 0.1; ‘ ’ 1.

## DISCUSSION

Several phylogeographic studies have revealed spatial structure in the distribution of rhizobia (Silva *et al*. 2005; Cao *et al*. 2017; Stefan *et al*. 2018), while others have found no such patterns (Fierer and Jackson 2006). Where found, spatial patterns were shown to be influenced by edaphic and climatic factors (Zahran 1999; Bissett *et al*. 2010; Stefan *et al*. 2018). It is known that common bean responds erratically to inoculation (Martínez-Romero 2003). Characterizing biogeographic patterns in rhizobia, in addition to evaluating the nodulation status, symbiotic effectiveness of soil rhizobia and response to N-fertilization, is of direct relevance to the development of inoculation strategies, since it can identify locations where the application of inoculants may be needed. In other words, detecting the genetic structure of indigenous rhizobia and causes of this structure across environments can help to identify the reasons for erratic inoculation responses. To determine the spatial patterns of bean-nodulating rhizobia, we first studied phylogenetic relationship of previously and currently isolated local strains and subsequently evaluated their biogeographic patterns. Phylogenetically, distinct *Rhizobium* genospecies clusters were determined. Housekeeping genes allowed us to identify eight genospecies of *Rhizobium* lineages, while *nodC* gene determined two main clusters that entirely belonged to genus *Rhizobium*. The *Rhizobium* species *R. tropici*, which is commonly used as an inoculant for beans in Africa and Latin America, was rarely detected in Ethiopian soils. Phylogeographic analysis did not reveal any spatial patterns among strains, but indicated differences between the strain collections that were linked to two species. The first species belonged to *R. aethiopicum*, while second belonged to *R. sophoriradicis*, each of which correspond to previous and current strain collections, respectively. The difference between the strain collections was influenced by soil pH, in which the *R. aethiopicum* strains occur more frequently in soils with high pH.

### Phylogenetics

Analysis of 16S rRNA assigned the strains to either *Rhizobium* or *Agrobacterium* lineages without clear delineation, confirming that it has poor strength to resolve the strains at species level (Vinuesa *et al*. 2005). MLSA that also included the 16S rRNA gene similarly assigned the strains into *Rhizobium* and *Agrobacterium* genospecies (clusters) with higher resolution. Common bean is a promiscuous host, able to form symbioses with diverse rhizobia (Martínez-Romero 2003), including >30 distinct species from different genera (Mwenda *et al*. 2018; Gunnabo *et al*. 2019; Huo *et al*. 2019). By contrast our results reveal that all of the strains nodulating common bean that we isolated from Ethiopian soils are limited to the single genus *Rhizobium*. Most of these strains belonged to the genospecies *R. etli* and *R. phaseoli*, while others belonged to minor groups *R. sophoriradicis*, *Rhizobium* sp. (also reported from beans grown in Kenya; Mwenda *et al*. 2018), *R. aethiopicum*, *R. chutanense* and undescribed genospecies *Rhizobium* sp. 1. The strains that were clustered in genospecies *R. phaseoli* were also closely related to *Rlp* strain CCGM1, which share a similar symbiotic gene *nodC* with that of *R. phaseoli*. The *Rlp* strains were previously reported from Ethiopia (Beyene *et al*. 2004; Aserse *et al*. 2012) and often from European soils (Pastor-Bueis *et al*. 2019). However, strains belonging to other genera such as *Methylobacterium* sp. strain AC72a and *Bradyrhizobium liaoningense* strain AC70 (Wolde-meskel *et al*. 2005) and *Pararhizobium giardinii* strain HBR21 (Aserse *et al*. 2012) were previously reported from root nodules of beans from Ethiopia. The *Methylobacterium* and *Bradyrhizobium* strains were isolated from non-agricultural soils. In centers of origin and diversification of common bean, the crop has been shown to associate with diverse species that belong to *R. phaseoli*, *R. etli* and *R. leguminosarum* sp., *R. leucaenae* and *R. tropici* (Ribeiro *et al*. 2013). Some of the *R. phaseoli*, *R. etli* and *R. leguminosarum* sp. groups were later reclassified as *R. ecuadorense* (Ribeiro *et al*. 2015) and *R. esperanzae* (Cordeiro *et al*. 2017) that are now part of the *leguminosarum–etli* group (Young *et al*. 2021).

In Tunisia, strains from genus *Ensifer*(*Sinorhizobium*) were found to be frequent in nodules of common bean in addition to the genus *Rhizobium* (Mhamdi *et al*. 2002; Mnasri *et al*. 2012). Environmental factors might shape the dominance of *Ensifer* in Tunisia where soils are often saline. Besides this, on a regional basis strains could become dominant symbionts of common bean as a result of coevolution or adaptation (Remigi *et al*. 2016), since legume genotypes can select symbiotic genotypes during the coadaptation process (Aguilar, Riva and Peltzer 2004; Li *et al*. 2016).

As indicated above, *R. etli* and *R. phaseoli* strains were the most dominant species, representing ∼70% of all isolates, consistent with previous reports (Aserse *et al*. 2012). However, Beyene *et al*. (2004) claimed that *R. leguminosarum* was the dominant species nodulating common bean in Ethiopia based on the analysis of multilocus enzyme electrophoresis (MLEE), while their analysis based on 16S rRNA revealed *R. etli* as a dominant strain. They concluded that transfer and recombination of 16S rRNA gene from *R. etli* to the local strains of *R. leguminosarum* must have taken place. Using MLSA analysis, several strains previously recognized as *R. leguminosarum* bv. *phaseoli* were reclassified as *R. etli* (Segovia, Young and Martinez-Romero 1993), *R. tropici* (Martinez-Romero *et al*. 1991) and *R. phaseoli* (Ramírez-Bahena *et al*. 2008). Hence, the *R. leguminosarum* strains described previously from Ethiopia could relate to one of these re-described species. *Rhizobium etli* and *Rhizobiumphaseoli* are believed to have originated along with common beans on the Americas, where they are the predominant strains found in its root nodules (Díaz-Alcántara *et al*. 2014). *Rhizobium etli* was shown to be carried on the testa of the bean seed (Perez-Ramirez *et al*. 1998), which has been proposed as a possible means that the species became distributed worldwide (Rodiño *et al*. 2010). None of the current isolates belonged to *R. aethiopicum* that was recently described from Ethiopia (Aserse *et al*. 2017). Likewise, the previous collection did not contain the genospecies *R. sophoriradicis*, found repeatedly in the current collection, reflecting the effect of sampling as reported previously (Alberton, Kaschuk and Hungria 2006).

There are other species of rhizobia that nodulate common bean, among which *R. tropici* is used in inoculants in Latin America and Africa and known to be genetically stable and adapted to adverse environments such as acidic soils and high temperatures (Martinez-Romero *et al*. 1991; Gomes, Ormeño-orrillo and Hungria 2015). We trapped only a single strain NAE165 that belonged to *R. tropici* species. This strain was not included in the MLSA phylogeny as we were not able to amplify its *rpoB* gene. The recovery of NAE165 strain from common bean root nodules confirms the occurrence in Ethiopian soils, albeit at low frequencies (Beyene *et al*. 2004; Aserse *et al*. 2012, 2013). Strains of *R. leucaenae* and *Pararhizobium giardinii* also occur only sporadically in root nodules of beans since only a single representative strain was described for each of these species (Aserse *et al*. 2012). By contrast, *R. tropici* strains were frequent in root nodules of beans growing in acid soils of Kenya (Anyango *et al*. 1995; Mwenda *et al*. 2018), indicating that they are favored by low soil pH.

Although two of the largest symbiovar clades identified by the *nodC* phylogeny (Table 3; Fig. 3) were dominated by strains of a single genospecies, all symbiovars contained members of different genospecies reflecting either phylogenetic uncertainty due to low sequence divergence or plasmid exchange between genospecies. It is well established that symbiotic genes show horizontal gene transfer, causing phylogenetic incongruencies between symbiotic and chromosomal genes (Young and Haukka 1996). Because rhizobial populations can adapt to distinct soil habitats, it is possible that they harbor a distinct set of chromosomal housekeeping loci, yet share a pool of identical plasmids that enables them to nodulate the same host legume across habitats (Wernegreen and Riley 1999; Zahran 2017). Such symbiovars unite a group of strains that share a genetic module conferring a distinct symbiotic phenotype (Laguerre, Mazurier and Amarger 1992). In the current case, the *nodC* phylogeny formed five different symbiovars. These symbiovars define the host specificity (Rogel *et al*. 2011; Lindstrom and Mousavi 2019). Strains in each of the symbiovars we identified have different chromosomal gene backgrounds and belong to different bacterial lineages confirming that symbiotic genes are transferred among the common bean-nodulating rhizobia (Rogel *et al*. 2011). Our cross-tabulation analysis of symbiovars and genospecies supported the conclusion that symbiovars belong to different chromosomal lineages. For instance, strains in symbiovar cluster I (Fig. 3) were related to reference strains that have different chromosomal gene background such as *R. ecuadorense*, *R. phaseoli*, *R. vallis* and *R. etli* (Table 3) and can be considered as *phaseoli* symbiovars (Rogel *et al*. 2011). The other important and large group (cluster III; Fig. 3) comprised strains from *R. aethiopicum* and *R. sophoriradicis* strains and can be considered as *R. aethiopicum* symbiovar phaseoli or *R. sophoriradicis* symbiovar phaseoli. The *R. sophoriradicis*-related symbiovars were also identified from common bean grown in China and Kenya (Mwenda *et al*. 2018; Tong *et al*. 2018), showing the species has a wide geographic distribution. The co-occurrence and coadaptation of *R. etli* and *R. phaseoli* strains with host legumes in a given locality may have helped the strains to have higher frequencies and occur sympatrically.

### Genetic diversity

The *nodC* and MLSA phylogenies revealed that genetic diversity of Ethiopian bean rhizobia (local strains) reflects only a sub-set of common bean-nodulating rhizobia, as a number of species known to nodulate beans, such as *R. freirei*, *R. mesoamericanum*, *R. lusitanum*, etc., were not detected. On the other hand, the nucleotide diversity observed in the previous collection of Ethiopian strains was only marginally lower than that observed for the set of bean-nodulating reference strains, reflecting considerable diversity within clades (Table 2). Perhaps the latter finding is the reason that earlier studies claimed exceptionally high genetic diversity among Ethiopian rhizobia (Beyene *et al*. 2004; Aserse *et al*. 2012). The large genetic diversity of common bean landraces reported from Ethiopia (Asfaw *et al*. 2009) could thus be linked to the large genetic diversity of rhizobia reported from the country so far.

### Phylogeography and genetic structure

We found no evidence of spatial patterns in rhizobial diversity or distribution of clades. This lack of spatial structure is mostly caused by the widespread distribution of *R. etli* and *R. phaseoli*, which occurs sympatrically and at high frequencies throughout the country. This distribution may reflect their broad adaptation to soil conditions as well as coevolution with their host plant (Aguilar, Riva and Peltzer 2004), which was introduced to the country between the sixteenth and seventeenth centuries (Wortmann *et al*. 1998; Asfaw *et al*. 2009). It is likely that the widely distributed genospecies and associated symbiovars reflect introductions together with common bean seed, whereas a rarer genospecies such as *R. aethiopicum* represents a local strain that obtained its symbiotic genes by horizontal gene transfer (Aserse *et al*. 2012). Recently, strains related to *R. aethiopicum* were recovered from root nodules of Berseem clover grown in Egypt (Youseif *et al*. 2021). Based on *nodC* gene analysis, they belong to a symbiovar trifolii and had superior nodulation and nitrogen fixation with clover. This suggests that the *R. aethiopicum* strains may have a different indigenous host plant other than common bean in Ethiopia and have gained symbiotic genes from *R. etli* through horizontal gene transfer. *Rhizobium etli* strains were shown to be carried on bean seed testa (Perez-Ramirez *et al*. 1998) to which *R. aethiopicum* strains are closely related (Aserse *et al*. 2017). Further, host range test of the *R. aethiopicum* strains on various indigenous and other legume plants of Ethiopia is needed to establish whether it is a microsymbiont of another legume species in Ethiopia. Our finding of a lack of phylogeographic pattern among common bean rhizobia contrasts with previous reports of strong patterns associated with environmental and climatic factors (Silva *et al*. 2005; Cao *et al*. 2017; Stefan *et al*. 2018). On the other hand, we observed significant clade differentiation between the two strain collections. The genospecies *R. aethiopicum* was associated with relatively alkaline soils within the sample suggesting that studies covering a wider range of environments may yet reveal relationships between edaphic factors such as pH and genospecies. At the level of symbiovar, however, the complete lack of genetic structure suggests that there is little association between symbiotic compatibility and geography. This finding agrees with previous work that found little or no biogeographic variation among rhizobia (Young, Demetriou and Apte 1987) but contrasts with several other studies that reported allele frequency differences among geographically separated populations (Aguilar, Riva and Peltzer 2004). The latter suggests that sampling more strains at individual locations could provide improved power to detect weak degrees of genetic differentiation.

## CONCLUSION

In conclusion, we show that there is high genetic diversity within clades but less variation among overall rhizobia nodulating common bean growing in Ethiopia compared with that described from other parts of the world. The species *R. etli* and *R. phaseoli* predominate in the root nodules of bean and occur sympatrically in different locations. The *R. etli* genospecies is more diverse than the *R. phaseoli* genospecies among the Ethiopian bean rhizobia, suggesting either faster sequence divergence within the *R. etli* genospecies, random introduction of *R. etli* and *R. phaseoli*, or a genetic bottleneck in *R. phaseoli*. Other species recovered from Ethiopian soils belong to *R. etli* bv. *phaseoli*, *R. sophorae*,*R. chutanense* and *Agrobacterium*. Strains such as *R. tropici*, *R. leucaenae* and *P. giardinii* only occasionally occupy the root nodules of beans growing in Ethiopia. The distribution of these strains reveals no spatial patterns over biogeography at various levels of genetic hierarchy. The presence of *R. aethiopicum* is seemingly related to soil pH but the influence of pH on both rhizobia collections did not allow us to confirm this. Thus, sampling more strains across a wider geographic range would be required to confirm whether soil pH influences bean rhizobia distributions in Ethiopia.

## FUNDING

We thank the Bill and Melinda Gates Foundation for partnering in this research through a grant to Wageningen University to support the project N2Africa: Putting Nitrogen Fixation to Work for Smallholder Farmers in Africa (www.N2Africa.org).

## Supplementary Material

fiab046_Supplemental_File
