# Supplementary material for: Phylogeographic distribution of rhizobia nodulating common bean (Phaseolus vulgaris L.) in Ethiopia
Source: FEMS Microbiol Ecol. 2021 Mar 16;97(4):fiab046. doi: 10.1093/femsec/fiab046 (PMC8016211; doi:10.1093/femsec/fiab046)
Supplement: fiab046_Supplemental_File [file fiab046_supplemental_file.pdf]

Supplemental Materials

Table S1: Strain information and relatedness to type strains

*Strain source	Strain Name	HK Cluster	Sym cluster	HK cluster Related to	nodC cluster Related to	Cultivars, sites, coordinates, Altitude and pH of sites of strains collection					
						Cultivars	Site	Lat	Long	Alt	pH
Previous	R. phaseoli HBR10	I	V	*R. phaseoli* ATCC14482	*R. lentis* BLR27	Redwolayta	Ambokersha (SE)	6.7723	37.7567	1662.00	6.01
Previous	R. phaseoli HBR17	I	V	*R. phaseoli* ATCC14482	*R. lentis* BLR27	Redwolayta	Tugaweransa (SE)	6.9109	38.4356	1662.00	6.72
Current	NAE1	I	IV	*R. phaseoli* ATCC14482	*R. phaseoli* ATCC14482	Ebado	Dulancho-Belila	7.6775	38.2458	2030.00	5.77
Current	NAE101	I	IV	*R. phaseoli* ATCC14482	*R. phaseoli* ATCC14482	Nasir	Gacheno	7.0403	37.9192	1863.00	6.00
Current	NAE103	I	IV	*R. phaseoli* ATCC14482	*R. phaseoli* ATCC14482	Ebado	Adilo	7.2039	37.9922	1922.00	5.85
Current	NAE109	I	IV	*R. phaseoli* ATCC14482	*R. phaseoli* ATCC14482	Hawassa Dume	Shanto-Katama	7.0264	37.8519	1935.00	6.40
Current	NAE14	I	IV	*R. phaseoli* ATCC14482	*R. phaseoli* ATCC14482	Ebado	Waja-Shoya	6.8597	37.7108	1903.99	6.02
Current	NAE153	I	IV	*R. phaseoli* ATCC14482	*R. phaseoli* ATCC14482	Ebado	Chulise	5.6006	37.8922	1664.00	5.59
Current	NAE174	I	IV	*R. phaseoli* ATCC14482	*R. phaseoli* ATCC14482	Nasir	Adilo	7.2039	37.9922	1922.00	5.85
Current	NAE179	I	IV	*R. phaseoli* ATCC14482	*R. phaseoli* ATCC14482	Ebado	Shanto-Katama	7.0264	37.8519	1935.00	6.40
Current	NAE187	I	IV	*R. phaseoli* ATCC14482	*R. phaseoli* ATCC14482	Hawassa Dume	Dulancho-Belila	7.6775	38.2458	2030.00	5.77
Current	NAE193	I	IV	*R. phaseoli* ATCC14482	*R. phaseoli* ATCC14482	Hawassa Dume	Chulise	5.6006	37.8922	1664.00	5.59
Current	NAE28	I	IV	*R. phaseoli* ATCC14482	*R. phaseoli* ATCC14482	Hawassa Dume	Kele	5.8267	37.9394	1368.90	6.22
Current	NAE28a	I	IV	*R. phaseoli* ATCC14482	*R. phaseoli* ATCC14482	Hawassa Dume	Kele	5.8267	37.9394	1368.90	6.22
Current	NAE31	I	IV	*R. phaseoli* ATCC14482	*R. phaseoli* ATCC14482	Field standing	Dulancho-Belila	7.6775	38.2458	2030.00	5.77
Current	NAE45	I	IV	*R. phaseoli* ATCC14482	*R. phaseoli* ATCC14482	Ebado	Hobichaka	7.2644	37.7431	2324.00	5.54
Current	NAE49	I	IV	*R. phaseoli* ATCC14482	*R. phaseoli* ATCC14482	Hawassa Dume	Shanto-Katama	7.0264	37.8519	1935.00	6.40
Current	NAE57	I	IV	*R. phaseoli* ATCC14482	*R. phaseoli* ATCC14482	Ebado	Menzo	7.5358	38.1789	1857.52	5.65
Current	NAE93	I	IV	*R. phaseoli* ATCC14482	*R. phaseoli* ATCC14482	Field standing	Tore-Badiya	5.8903	38.1519	1677.88	5.80
Previous	R. phaseoli HBR1	I	IV	*R. phaseoli* ATCC14482	*R. phaseoli* ATCC14482	Redwolayta	Genetamecha (SE)	6.0266	37.4507	1401.00	6.87
Previous	R. phaseoli HBR11	I	IV	*R. phaseoli* ATCC14482	*R. phaseoli* ATCC14482	Redwolayta	Harar (EE)	9.3821	42.1319	1417.00	6.96
Previous	R. phaseoli HBR13	I	IV	*R. phaseoli* ATCC14482	*R. phaseoli* ATCC14482	Redwolayta	Harar (EE)	9.3300	42.1192	1417.00	7.13
Previous	R. phaseoli HBR18	I	IV	*R. phaseoli* ATCC14482	*R. phaseoli* ATCC14482	Redwolayta	Adello (SE)	7.2262	38.0330	1662.00	7.13

Previous	R. phaseoli HBR20	I	IV	*R. phaseoli* ATCC14482	*R. phaseoli* ATCC14482	Redwolayta	Leku	6.9214	38.4581	1662.00	6.80
Previous	R. phaseoli HBR53	I	IV	*R. phaseoli* ATCC14482	*R. phaseoli* ATCC14482	Redwolayta	Dembi (WE)	8.0744	36.4575	1637.00	6.00
Current	NAE186	I	I	*R. phaseoli* ATCC14482	*R. phaseoli* Brasil5	Ebado	Dulancho-Belila	7.6775	38.2458	2030.00	5.77
Current	NAE199	I	UX	*R. phaseoli* ATCC14482	UX_22	Ebado	Adilo	7.2039	37.9922	1922.00	5.85
Current	NAE38	I	UX	*R. phaseoli* ATCC14482	UX_24	Nasir	Otomalo	5.4847	37.8692	1828.00	5.74
Current	NAE26	I	UX	*R. phaseoli* ATCC14482	X_8	Ebado	Dulancho-Belila	7.6775	38.2458	2030.00	5.77
Current	NAE26a	I	UX	*R. phaseoli* ATCC14482	X_8	Ebado	Dulancho-Belila	7.6775	38.2458	2030.00	5.77
Current	NAE138	I		*R. phaseoli* ATCC14482	NA	Ebado	Adilo	7.2039	37.9922	1922.00	5.85
Current	NAE145	I		*R. phaseoli* ATCC14482	NA	Hawassa Dume	Kele	5.8267	37.9394	1368.90	6.22
Current	NAE39	I		*R. phaseoli* ATCC14482	NA	Nasir	Grumo-Woyde	6.9319	37.7289	1948.00	4.86
Current	NAE71	I		*R. phaseoli* ATCC14482	NA	Hawassa Dume	Kele	5.8267	37.9394	1368.90	6.22
Current	NAE127	II	III	*R. sophoriradicis* CCBAU03470	*R. sophoriradicis* CCBAU03470	Nasir	Suluko	5.6533	37.8964	1573.00	5.75
Current	NAE130	II	III	*R. sophoriradicis* CCBAU03470	*R. sophoriradicis* CCBAU03470	Ebado	Menzo	7.5358	38.1789	1857.52	5.65
Current	NAE167	II	III	*R. sophoriradicis* CCBAU03470	*R. sophoriradicis* CCBAU03470	Hawassa Dume	Kele	5.8267	37.9394	1368.90	6.22
Current	NAE183	II	III	*R. sophoriradicis* CCBAU03470	*R. sophoriradicis* CCBAU03470	Ebado	Gacheno	7.0369	37.9172	1887.34	5.54
Current	NAE27	II	III	*R. sophoriradicis* CCBAU03470	*R. sophoriradicis* CCBAU03470	Hawassa Dume	Gacheno	7.0369	37.9172	1887.34	6.01
Current	NAE34	II	III	*R. sophoriradicis* CCBAU03470	*R. sophoriradicis* CCBAU03470	Ebado	Adilo	7.2039	37.9922	1922.00	5.85
Current	NAE47	II	III	*R. sophoriradicis* CCBAU03470	*R. sophoriradicis* CCBAU03470	Ebado	Gacheno	7.0369	37.9172	1887.34	6.01
Previous	R. aethiopicum HBR3	III	II	X_6	*R. sophorae* CCBAU03386	Redwolayta	Selamber (SE)	6.4935	37.4699	1401.00	6.50
Previous	R. aethiopicum HBR23	III	III	X_6	*R. sophoriradicis* CCBAU03470	Redwolayta	Addis (CE)	9.0331	38.7628	2327.00	7.12
Previous	R. aethiopicum HBR26	III	III	X_6	*R. sophoriradicis* CCBAU03470	Redwolayta	Kurfana Soloke (CE)	8.5972	39.3804	1661.00	6.71
Previous	R. aethiopicum HBR31	III	III	X_6	*R. sophoriradicis* CCBAU03470	Redwolayta	Mieaso (EE)	7.2237	40.7088	1599.00	7.05
Previous	R. aethiopicum HBR50	III	III	X_6	*R. sophoriradicis* CCBAU03470	Redwolayta	Abeshegie Jejeba (SE)	8.2700	37.7279	1808.00	7.90
Previous	R. aethiopicum HBR7	III	III	X_6	*R. sophoriradicis* CCBAU03470	Redwolayta	Gata (SE)	5.5390	37.4144	1401.00	8.78
Previous	R. etli HBR14	IV	V	*R. etli* CFN42	*R. lentis* BLR27	Redwolayta	Shaya (SE)	7.0336	37.9097	1662.00	6.88
Previous	R. etli HBR19	IV	IV	*R. etli* CFN42	*R. phaseoli* ATCC14482	Redwolayta	Awelgama-sitalo (SE)	9.2404	38.5126	2641.00	6.41
Current	NAE102	IV	I	*R. etli* CFN42	*R. phaseoli* Brasil5	Ebado	Adilo	7.2039	37.9922	1922.00	5.85
Current	NAE112	IV	I	*R. etli* CFN42	*R. phaseoli* Brasil5	Ebado	Gacheno	7.0403	37.9192	1863.00	6.00

Current	NAE134	IV	I	*R. etli* CFN42	*R. phaseoli* Brasil5	Hawassa Dume	Shanto-Katama	7.0264	37.8519	1935.00	6.40
Current	NAE175	IV	I	*R. etli* CFN42	*R. phaseoli* Brasil5	Hawassa Dume	Grumo-Woyde	6.9319	37.7289	1948.00	4.86
Current	NAE177	IV	I	*R. etli* CFN42	*R. phaseoli* Brasil5	Hawassa Dume	Shanto-Katama	7.0264	37.8519	1935.00	6.40
Current	NAE185	IV	I	*R. etli* CFN42	*R. phaseoli* Brasil5	Hawassa Dume	Kele	5.8267	37.9394	1368.90	6.22
Current	NAE190	IV	I	*R. etli* CFN42	*R. phaseoli* Brasil5	Ebado	Adilo	7.2039	37.9922	1922.00	5.85
Current	NAE198	IV	I	*R. etli* CFN42	*R. phaseoli* Brasil5	Ebado	Gututo	6.7544	37.7731	1806.00	5.74
Current	NAE4	IV	I	*R. etli* CFN42	*R. phaseoli* Brasil5	Ebado	Chulise	5.6006	37.8922	1664.00	5.59
Current	NAE5	IV	I	*R. etli* CFN42	*R. phaseoli* Brasil5	Ebado	Myo-Kote	6.8919	37.8492	2105.35	5.42
Current	NAE50	IV	I	*R. etli* CFN42	*R. phaseoli* Brasil5	Hawassa Dume	Shanto-Katama	7.0264	37.8519	1935.00	6.40
Current	NAE51	IV	I	*R. etli* CFN42	*R. phaseoli* Brasil5	Ebado	Chulise	5.6006	37.8922	1664.00	5.59
Current	NAE78	IV	I	*R. etli* CFN42	*R. phaseoli* Brasil5	Hawassa Dume	Dulancho-Belila	7.6775	38.2458	2030.00	5.77
Previous	R. etli HBR2	IV	I	*R. etli* CFN42	*R. phaseoli* Brasil5	Redwolayta	Goho (SE)	5.3980	37.1526	1504.00	6.61
Previous	R. etli HBR24	IV	I	*R. etli* CFN42	*R. phaseoli* Brasil5	Redwolayta	Addis (CE)	8.8333	38.8333	1661.00	7.30
Previous	R. etli HBR4	IV	I	*R. etli* CFN42	*R. phaseoli* Brasil5	Redwolayta	Kemogerbi (CE)	7.9398	38.7066	2883.00	7.90
Previous	R. etli HBR5	IV	I	*R. etli* CFN42	*R. phaseoli* Brasil5	Redwolayta	Mechela (SE)	5.3638	37.3669	1504.00	6.72
Previous	R. etli HBR51	IV	I	*R. etli* CFN42	*R. phaseoli* Brasil5	Redwolayta	Amba 13 (WE)	9.8195	34.6918	827.00	6.00
Current	NAE135	IV	II	*R. etli* CFN42	*R. sophorae* CCBAU03386	Ebado	Gacheno	7.0403	37.9192	1863.00	6.00
Current	NAE149	IV	II	*R. etli* CFN42	*R. sophorae* CCBAU03386	Field standing	Lemo	5.4228	37.8692	1878.83	5.65
Current	NAE201	IV	II	*R. etli* CFN42	*R. sophorae* CCBAU03386	Field standing	Lemo	5.4239	37.8731	1916.64	6.44
Current	NAE36	IV	II	*R. etli* CFN42	*R. sophorae* CCBAU03386	Nasir	Shanto-Katama	7.0264	37.8519	1935.00	6.40
Current	NAE111	IV	III	*R. etli* CFN42	*R. sophoriradicis* CCBAU03470	Ebado	Gututo	6.7544	37.7731	1806.00	5.74
Current	NAE113	IV	III	*R. etli* CFN42	*R. sophoriradicis* CCBAU03470	Nasir	Gacheno	7.0403	37.9192	1863.00	6.00
Current	NAE156	IV	III	*R. etli* CFN42	*R. sophoriradicis* CCBAU03470	Ebado	Chulise	5.6006	37.8922	1664.00	5.59
Current	NAE173	IV	III	*R. etli* CFN42	*R. sophoriradicis* CCBAU03470	Nasir	Suluko	5.6533	37.8964	1573.00	5.75
Current	NAE200	IV	III	*R. etli* CFN42	*R. sophoriradicis* CCBAU03470	Ebado	Waleya	5.5956	37.9067	1579.00	5.73
Current	NAE204	IV	III	*R. etli* CFN42	*R. sophoriradicis* CCBAU03470	Field standing	Lemo	5.4239	37.8731	1916.64	6.44
Current	NAE46	IV	III	*R. etli* CFN42	*R. sophoriradicis* CCBAU03470	Ebado	Hobichaka	7.2644	37.7431	2324.00	5.54
Current	NAE80	IV	III	*R. etli* CFN42	*R. sophoriradicis* CCBAU03470	Ebado	Gututo	6.7544	37.7731	1806.00	5.74

Current	NAE3	IV	UX	*R. etli* CFN42	UX_23	Ebado	Gututo	6.7544	37.7731	1806.00	5.74
Current	NAE189	IV		*R. etli* CFN42	NA	Ebado	Dulancho-Belila	7.6775	38.2458	2030.00	5.77
Current	NAE191	U	I	X_24	*R. phaseoli* Brasil5	Nasir	Shanto-Katama	7.0264	37.8519	1935.00	6.40
Current	NAE120	U	II	X_24	*R. sophorae* CCBAU03386	Field standing	Lemo	5.4228	37.8692	1878.83	5.65
Previous	R. sp. HBR42	V	IV	*R. ecuadorense* CNPSo671	*R. phaseoli* ATCC14482	Redwolayta	Efabas (EE)	9.1047	40.9606	1599.00	7.11
Current	NAE164	V	III	*R. ecuadorense* CNPSo671	*R. sophoriradicis* CCBAU03470	Nasir	Shanto-Katama	7.0264	37.8519	1935.00	6.40
Current	NAE166	V	III	*R. ecuadorense* CNPSo671	*R. sophoriradicis* CCBAU03470	Hawassa Dume	Myo-Kote	6.8919	37.8492	2105.35	5.42
Previous	R. sp. HBR22	V	III	*R. ecuadorense* CNPSo671	*R. sophoriradicis* CCBAU03470	Redwolayta	Addis (CE)	9.0331	38.7628	2327.00	7.47
Current	NAE182	V	UX	*R. ecuadorense* CNPSo671	UX_20	Field standing	Tore-Badiya	5.9131	38.1628	1691.57	6.01
Current	NAE44	V		*R. ecuadorense* CNPSo671	NA	Ebado	Hobichaka	7.2644	37.7431	2324.00	5.54
Current	NAE60a	V		*R. ecuadorense* CNPSo671	NA	NA	NA	NA	NA	NA	NA
Current	NAE136	VI	III	X_29	*R. sophoriradicis* CCBAU03470	Nasir	Dulancho-Belila	7.6775	38.2458	2030.00	5.77
Current	NAE154	VI	III	X_29	*R. sophoriradicis* CCBAU03470	Ebado	Adilo	7.2039	37.9922	1922.00	5.85
Current	NAE23	VI	III	X_29	*R. sophoriradicis* CCBAU03470	Ebado	Adilo	7.2039	37.9922	1922.00	5.85
Current	NAE52	VII	NA	*Agrobacterium tumefaciens* C58	NA	Hawassa Dume	Shanto-Katama	7.0264	37.8519	1935.00	6.40
Current	NAE55	VII	NA	*Agrobacterium tumefaciens* C58	NA	Nasir	Gacheno	7.0403	37.9192	1863.00	6.00
Current	NAE55a	VII	NA	*Agrobacterium tumefaciens* C58	NA	Nasir	Gacheno	7.0403	37.9192	1863.00	6.00
Previous	Agrobacterium sp. HBR33	VII	NA	*Agrobacterium tumefaciens* C58	NA	Redwolayta	Gulfa (EE)	9.1566	40.8053	1599.00	6.95
Previous	Agrobacterium sp. HBR52	VII	NA	*Agrobacterium tumefaciens* C58	NA	Redwolayta	Adamitulu (CE)	7.1347	38.1022	2883.00	7.31
Previous	Agrobacterium sp. HBR75	VII	NA	*Agrobacterium tumefaciens* C58	NA	Redwolayta	Efabas (EE)	9.1796	41.1077	1599.00	6.86
Previous	Agrobacterium tumefaciens HBR45	VII	NA	*Agrobacterium tumefaciens* C58	NA	Redwolayta	Terkam Feta (EE)	9.1797	41.1081	2327.00	6.95
Previous	Agrobacterium tumefaciens HBR78	VII	NA	*Agrobacterium tumefaciens* C58	NA	Redwolayta	Kobo (EE)	9.3648	41.6610	1599.00	7.08
Previous	R. giardinii HBR21		NA	*R. giardinii* bv. *giardinii* H152	*R. giardinii* bv. *giardinii* H152	Redwolayta	Addis (CE)	9.0331	38.7628	2327.00	7.15
Current	NAE165		VI	NA	*R. leucaenae* CFN299	Nasir	Shanto-Katama	7.0264	37.8519	1935.00	6.40
Current	R. sp. NAK103		VI	NA	*R. leucaenae* CFN299	Bean	Kenya	NA	NA	NA	NA
Current	R. sp. NAK91		VI	NA	*R. leucaenae* CFN299	Bean	Kenya	NA	NA	NA	NA
Previous	R. leucaenae HBR12		VI	NA	*R. leucaenae* CFN299	Redwolayta	Wachigoesho (SE)	6.7721	37.6665	1662.00	6.02
Current	NAE2		IV	NA	*R. phaseoli* ATCC14482	Ebado	Dulancho-Belila	7.6775	38.2458	2030.00	5.77
Current	NAE76		IV	NA	*R. phaseoli* ATCC14482	Nasir	Chulise	5.6006	37.8922	1664.00	5.59

Current	NAE82	IV	NA	*R. phaseoli* ATCC14482	Ebado	Adilo	7.2039	37.9922	1922.00	5.85
Previous	R. phaseoli HBR9	IV	NA	*R. phaseoli* ATCC14482	Redwolayta	Alawla (SE)	8.3104	37.6295	1808.00	7.90
Current	NAE122	I	NA	*R. phaseoli* Brasil5	Nasir	Suluko	5.6533	37.8964	1573.00	5.75
Current	NAE124	I	NA	*R. phaseoli* Brasil5	Nasir	Otomalo	5.4994	37.8850	1747.92	6.14
Current	NAE203	II	NA	*R. sophorae* CCBAU03386	Field standing	Lemo	5.4239	37.8731	1916.64	6.44
Current	NAE181	III	NA	*R. sophoriradicis* CCBAU03470	Hawassa Dume	Billa	5.5578	37.9097	1591.25	5.78
Current	NAE205	III	NA	*R. sophoriradicis* CCBAU03470	Nasir	Suluko	5.6533	37.8964	1573.00	5.75
Current	NAE30	III	NA	*R. sophoriradicis* CCBAU03470	Ebado	Chulise	5.6006	37.8922	1664.00	5.59
Current	NAE83	III	NA	*R. sophoriradicis* CCBAU03470	Nasir	Shanto-Katama	7.0264	37.8519	1935.00	6.40
Previous	R. sp. HBR79	III	NA	*R. sophoriradicis* CCBAU03470	NA	NA	NA	NA	NA	NA
Current	NAE19	UX	NA	UX_21	Nasir	Suluko	5.6533	37.8964	1573.00	5.88
Current	NAE216	UX	UX_25		Bean					

Where: ATCC14482 = *R. phaseoli*; Brasil5 = *R. phaseoli*; CCBAU03470 = *R. sophoriradicis*; CNPSo671 = R. *ecuadorense*; CFN42 = *R. etli*; CCBAU03386 = *R. sophorae*; H152 = *R. giardinii* bv. *giardinii* ; C58 = *Agrobacterium tumefaciens*; HK = Housekeeping genes; Sym = Symbiotic genes (nodC); Lat = Latitude; Long = longitude; Alt = Altitude; pH = Soil pH

*Strain sources indicate strains that were collected by this study (current) and strains that were previously collected from Ethiopia and published elsewhere (previous).

Table S2: List of primers and their PCR conditions

Loci	Primer and their target gene position	Primer sequence 5'-3'	PCR condition	References
16S rRNA	63F<br>1389R	CAG GCC TAA CAC ATG CAA GTC<br>ACG GGC GGT GTG TAC AAG	5 min 95^{0}C, 35x(30 sec 95^{0}C, 30 sec 55^{0}C, 1 min 72^{0}C), 7 min 72^{0}C	(Marchesi *et al.*, 1998)
nodC	nodCfor540 (544–566)<br>nodCrev1160 (1164–1184)	TGA TYG AYA TGG ART AYT GGC T<br>CGY GAC ARC CAR TCG CTR TTG	2 min 98°C, 34x(15 sec 98°C, 20 min 63°C, 20 sec 72°C), 5 min 72°C	(Aserse *et al.*, 2012)
nifH	nifH-1F (367-389)<br>nifH-1R (794-774)	GTC TCC TAT GAC GTG CTC GG<br>GCT TCC ATG GTG ATC GGG GT	5 min 95°C, 35x(30 sec 95°C, 30 sec 57 ^{o}C, 1 min 2°C), 7 min 72 ^{o}C	(Aserse *et al.*, 2012)
recA	recA-6F (16-31)<br>recA-555R (555-530)	CGK CTS GTA GAG GAY AAA TCG GTG GA<br>CGR ATC TGG TTG ATG AAG ATC ACCAT	10 min 95^{0}C, 35x(30 sec 95^{0}C, 45 sec 57^{0}C, 1 min 72^{0}C), 7 min 72^{0}C	(Aserse *et al.*, 2012)
rpoB	rpoB-83F (83-103)<br>rpoB-1061R (1081-1061)	CCT SAT CGA GGT TCA CAG AAG GC<br>AGC GTG TTG CGG ATA TAG GCG	5 min 95^{0}C, 3x(2 min 94^{0}C, 2 min 58^{0}C, 1 min 72^{0}C), 30x(0.30' 94^{0}C,1 min 58^{0}C, 1 min 72^{0}C), 5 min 72^{0}C	(Aserse *et al.*, 2012)
glnII	glnII-12F<br>glnII-689R	YAA GCT CGA CTA CAT YTC<br>TGC ATG CCS GAG CCG TTC CA	10 min 95^{0}C, 35x(30 sec 95^{0}C, 45 sec 57^{0}C, 1 min 72^{0}C), 7 min 72^{0}C	(Vinuesa *et al.*, 2005)
gyrB	gyrB343F<br>gyrB1043R	TTC GAC CAG AAY TCC TAY AAG G<br>AGC TTG TCC TTS GTC TGC G	5 min 95^{0}C, 3x(2' 94^{0}C, 2 min 58^{0}C, 1 min 72^{0}C), 30x(0.30' 94^{0}C,1 min 58^{0}C, 1 min 72^{0}C), 5 min 72^{0}C	(Martens *et al.*, 2008)

Table S3: Description of *Rhizobium* strain categories (hierarchies) that were used in biogeographic analysis

Category levels	Description
Nucleotide	Based on differences between nucleotide sequences
Locus	Loci (haplotypes) are defined as having identical nucleotide differences at individual loci. Haplotype distance is defined as the proportion of identical sequences across a set of loci.
Species	Species are defined here as strains belonging to the same monophyletic MLSA clade as an identical reference strain. Species dissimilarity refers to the 0, 1 indicator matrix indicating which strain belong to the same species. Species diversity was calculated using Simpson's and Shannon's diversity measures

Supplemental figures

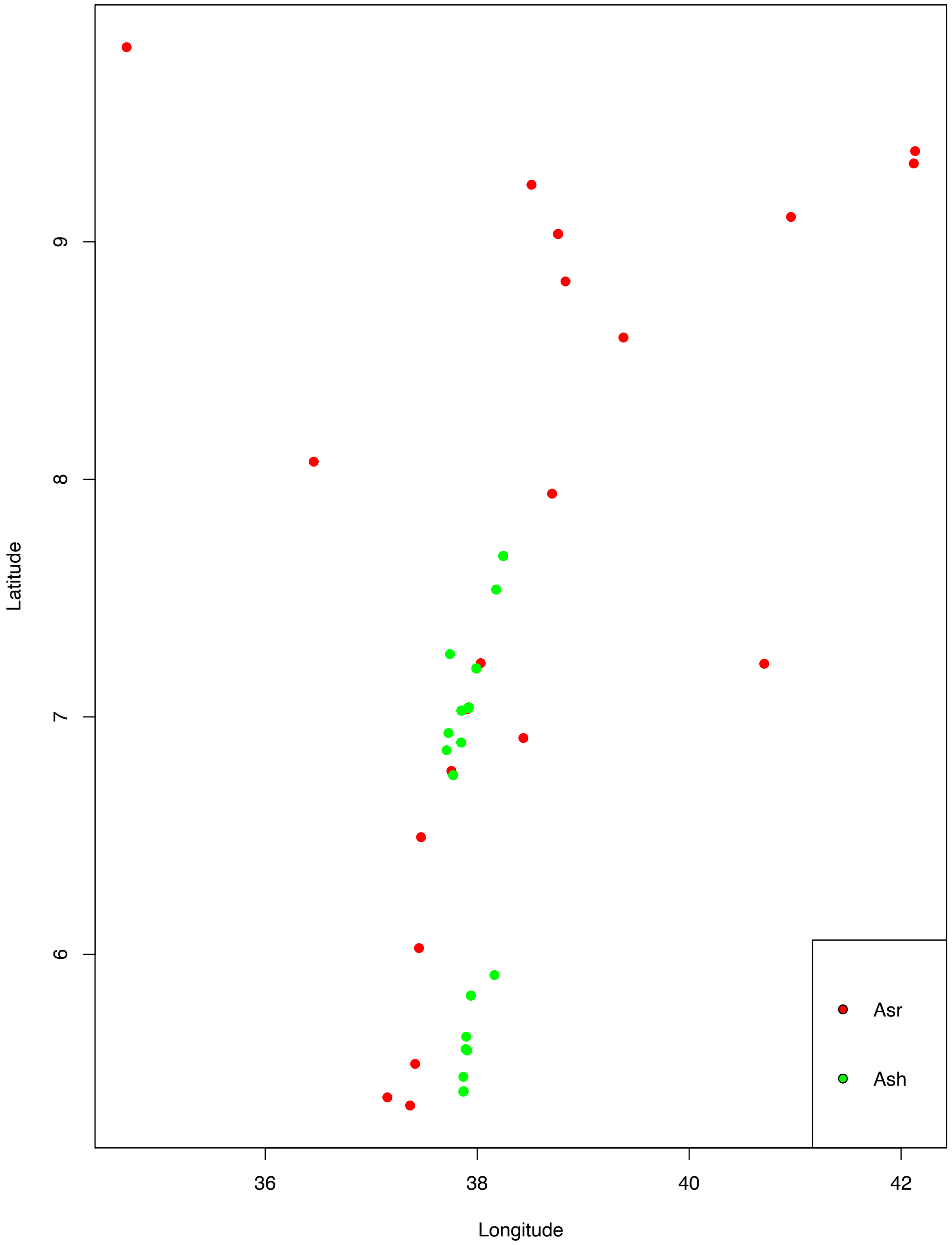

Figure S1: Distribution of sampling sites of rhizobia collections over geographic space. *Asr* stands for Aserse et al., 2012 that represents sampling points from where previous rhizobia collection were sampled, while *Ash* points represent sites from where the current rhizobia collection were ampled.

References:

Aserse, A.A., Räsänen, L.A., Assefa, F., Hailemariam, A., and Lindström, K. (2012) Phylogeny and genetic diversity of native rhizobia nodulating common bean (Phaseolus vulgaris L.) in Ethiopia. *Syst Appl Microbiol* **35**: 120–131.

Marchesi, J.R., Sato, T., Weightman, A.J., Martin, T.A., Fry, J.C., Hiom, S.J., et al. (1998) Design and evaluation of useful bacterium-specific PCR primers that amplify genes coding for bacterial 16S rRNA. *Appl Environ Microbiol* **64**: 795–799.

Martens, M., Dawyndt, P., Coopman, R., Gillis, M., De Vos, P., and Willems, A. (2008) Advantages of multilocus sequence analysis for taxonomic studies: A case study using 10 housekeeping genes in the genus Ensifer (including former Sinorhizobium). *Int J Syst Evol Microbiol* **58**: 200–214.

Vinuesa, P., Silva, C., Lorite, M.J., Izaguirre-Mayoral, M.L., Bedmar, E.J., and Martínez-Romero, E. (2005) Molecular systematics of rhizobia based on maximum likelihood and Bayesian phylogenies inferred from rrs, atpD, recA and nifH sequences, and their use in the classification of Sesbania microsymbionts from Venezuelan wetlands. *Syst Appl Microbiol* **28**: 702–716.
